# StaggR: an interactive R/Shiny application for planning and visualizing staggered experimental protocols

**DOI:** 10.12688/f1000research.168987.1

**Published:** 2025-09-15

**Authors:** Alex Michael Francette

**Affiliations:** 1 Department of Cell Biology and Physiology, Washington University in St Louis, St. Louis, Missouri, 63110, USA

**Keywords:** Workflow Optimization, Experimental Design, Reproducibility, High-Throughput Assays, Gantt Chart, Operations Research, R, Shiny

## Abstract

Biological experiments often require a series of precisely timed operations, and small variations in treatment can result in inconsistent or biased results. To handle multiple samples in parallel with precise temporal resolution, experimentalists may stagger treatments by initiating the workflow of one sample during the wait or incubation time of another. However, as the number of samples processed in parallel and the number of operations increase, it becomes increasingly difficult to identify and execute valid treatment regimens that permit the handling of each sample. To address this, I developed StaggR, an interactive web application that calculates and visualizes compatible staggering intervals for complex experimental workflows. This tool provides a user-friendly interface for defining protocol operations, durations, and wait times. It can automatically calculate the shortest possible conflict-free interval for initiating sample treatments, or allow users to simulate specific intervals to explore potential treatment regimens or bottlenecks. Using StaggR, users of any experience level can rapidly generate complete, color-coded experimental workflows, visualize these workflows in an easy-to-read chart, and execute them using a built-in timer displaying a treatment schedule with live updates. The experimental designs can be saved, shared, and re-imported, ensuring full reproducibility and user control. The application of StaggR is expected to expedite the design and throughput of complex experimental workflows while maximizing reproducibility.

## Introduction

Biological processes, such as pre-mRNA splicing, intracellular signaling cascades, and protein decay occur on rapid timescales ranging from microseconds to days ( Shamir et al., 2016). Therefore, to capture these dynamic events, research in fields such as pharmacology, biochemistry, and genomics often relies on complex and time-sensitive sample workflows. To execute protocols across multiple conditions with multiple replicates, an experimentalist will often stagger their treatments so that some samples can be handled during the hands-off times of others. When experiments are sensitive to small time variations, staggering treatments can be an effective tool for reducing experimental noise while simultaneously increasing productivity by reducing the time required to handle multiple samples. However, when calculating a valid, let alone minimized, staggering interval is a non-trivial cognitive task.

An excessively long staggering interval can make experiments too long to fit within the work period. However, reducing the interval too much can be overwhelming for an experimentalist to keep track of. Mishandling of samples due to poor experimental design can lead to compromised reproducibility and loss of valuable materials.

While simple protocols can be scheduled with a spreadsheet, complex workflows with numerous, non-uniform operations quickly become intractable to plan manually. Each additional time-sensitive task and sample added to the workflow creates a new potential scheduling conflict. Furthermore, some staggered experimental setups may have no conflict-free solutions. However, without extensive trial and error during planning, it is difficult to determine whether a schedule is possible. This challenge is a practical instance of a well-defined class of optimization problems in operations research, known as the Permutation Flow Shop Scheduling Problem (FSSP) ( Emmons, 2013). Approaches to address permutation FSSPs optimize how machines (in this case humans) perform sequential jobs (sample processing pipelines) which consist of multiple operations (sample processing steps) of discrete durations (“hands-on” times) with the constraint that operations must occur in the same order. In this case, there is a single operator responsible for all tasks who cannot be engaged with two tasks simultaneously. Typical solutions in this space are designed for machining applications. However, open-source tools to optimize the workflows for average users on the bench are lacking.

To address this problem intuitively, I present StaggR, an interactive platform that serves to optimize, simulate, and facilitate staggered experimental protocols. StaggR provides a graphical user interface for defining the number of samples to be processed in parallel and the key operations of a protocol, including hands-on durations, wait times, and realistic buffer periods for switching tasks. Based on this plan, StaggR will attempt to optimize the minimal valid staggering interval and provide a Gantt chart that visualizes a work schedule for processing the user-defined samples in parallel, a downloadable table with step-by-step instructions showing the chronological series of events that comprise the solved workflow, and a tool to dynamically track the progression of a workflow plan in real time to assist in the timely execution of operations. StaggR is a simple application designed to provide a clean, easy-to-use interface that quickly expands the capacity of experimentalists to explore and execute complex sample-processing pipelines.

## Methods

### Implementation

At the core of StaggR is a scheduling algorithm that uses an iterative search heuristic which simulates an experimental workflow involving multiple experiments with starting times offset by a defined time step automatically identified by the application or manually input by the user. In “automatic” mode, it begins with a minimal interval specified by the user through the “granularity” parameter and simulates the experiment. StaggR then checks for time points where the researcher is simultaneously occupied by the hands-on requirements for more than one task. If a conflict is detected, the interval is iteratively increased by the granularity value and the simulation is repeated until a conflict-free schedule is found. This ensures the shortest possible total experiment time without creating logistical impossibility. The algorithm also accounts for user-defined “buffer time” (e.g., 60 s), a short duration added to the end of the operation to model the time it takes to switch between tasks. Although more complex optimization methods exist, this iterative search heuristic was chosen for its straightforward implementation, computational speed for typical lab-scale problems, and its ability to guarantee a conflict-free, if not globally optimal, solution. StaggR runs the optimization by default. By enabling “manual” mode, the user may experiment with different staggering intervals to examine alternate treatment schedules.

**Figure 1.**
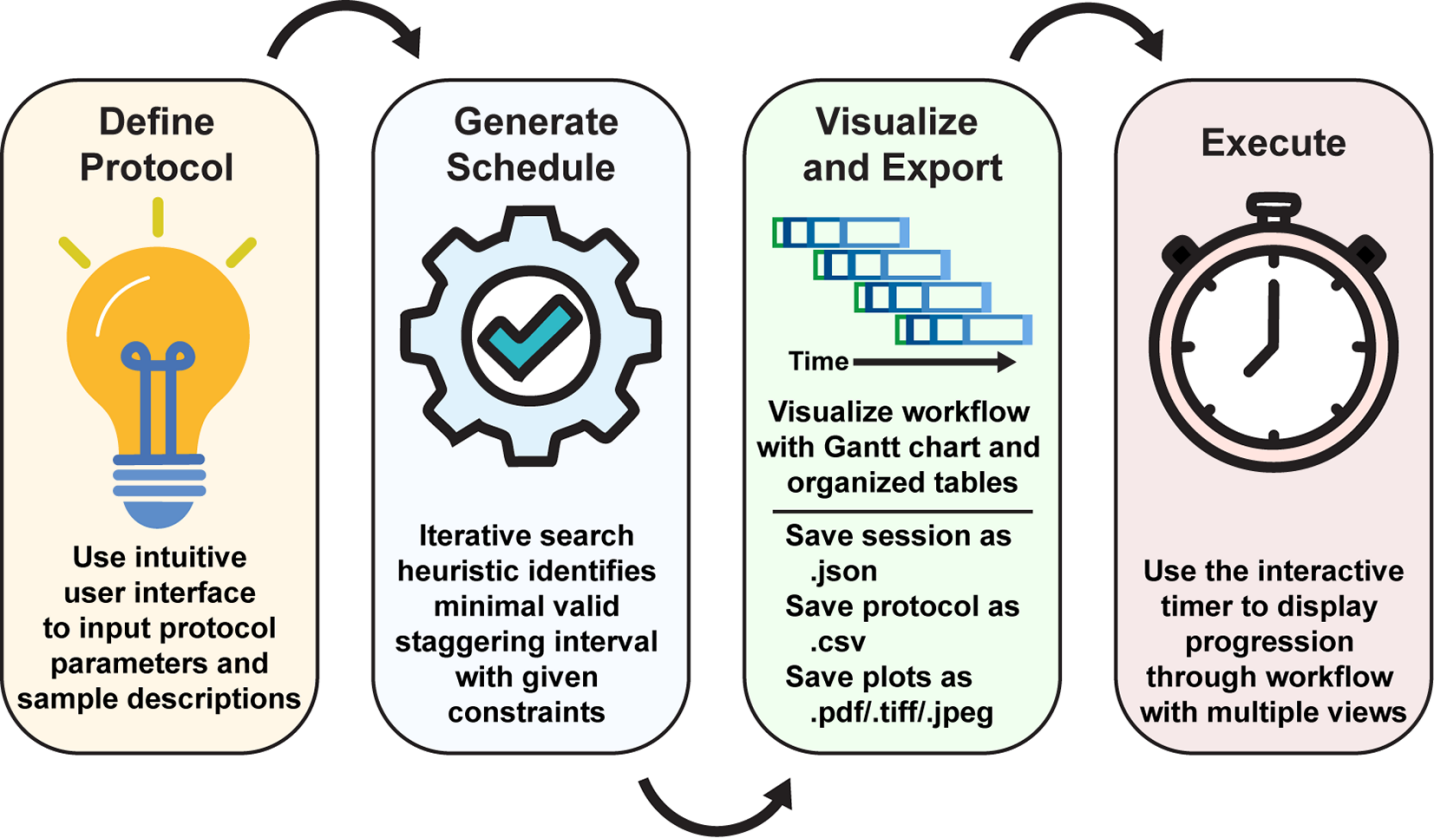
StaggR shiny app overview. Upon initialization of the application, the user inputs the experimental parameters and generates a schedule automatically, or with a given staggering interval. The workflow is then visualized with a Gantt chart and tables illustrating the staggered processing protocols for each sample or batch of samples. Using the “Live Time Course” tab, users can easily track the progression of their charted experimental workflow.

The core scheduling logic of StaggR was implemented in base R (v4.4.0), with data manipulation handled by the dplyr package ( Wickham et al., 2019). The interactive Gantt chart was generated using ggplot2 (v3.5.2) ( Wickham, 2011), and the user interface was built using shiny (v1.11.1) and shinyjs (v2.1.0) for dynamic reactivity. Data tables were rendered using the DT package (v0.33). The application is designed to run locally from the source code or be accessed as a hosted web service. The initial drafts of this code and manuscript were edited with the assistance of ChatGPT o3 (OpenAI) and Gemini 2.5 Pro (Google). All panels outside of Figure 1 are derived from screenshot images natively generated using the StaggR app.

### Operation

The StaggR interface is divided into a control sidebar for the parameter input and a main panel for visualizing the output. The workflow is described below ( Figure 1).

1. **Define Protocol** - The user first provides the following information to the scheduler:
  • **Sample descriptions**: The number and names of samples used for the protocol.
  • **Operation descriptions**: The number and names of the operations used in the protocol. Each operation is an action or a series of actions that require constant attention.
  • **Operation duration**: The hands-on duration in minutes for each operation. This is the maximum time allotted to an individual operation to take place. This parameter may be shorter for a more experienced experimentalist or longer for trainees new to the protocol. For the best results, it is recommended that inexperienced users perform a dry run for each operation to obtain a better understanding of their individual pacing.
  • **Inter-operation interval**: The “wait time” that occurs between the end of one operation and the beginning of the next (e.g., incubation, or duration of spin in a centrifuge).
  The user may additionally utilize the “Time Course Helper” to easily insert a simple time series by first choosing an anchoring operation (e.g., the start of a drug treatment), then defining an operation to be repeated (e.g., harvest cells) and lastly identifying the time points to enact the repeating operation (e.g., 5, 15, 30, and 60 min). All parameters may be saved and edited externally for re-uploading if desired.
  (Optional) Additional Parameters - StaggR provides additional flexibility in the planning through additional parameters that may be modified but are populated with convenient values by default.
  • **Buffer time**: The user may specify a defined “buffer time (default 60 s)” as a constant duration to allow switching between tasks.
  • **Optimizer granularity**: The user can define the lengths of the time steps for which the optimizer checks for conflict. This value will also be used as the first test interval. A smaller granularity often leads to a solution closer to the optimal solution, but requires a longer computation time. Adjust according to the time scale of the workflow.
  • **Graphical parameters**: The user can also modulate several features of the output displays including the colors of each operation used for visualization, a base color to generate a spread of colors for samples, and the X-axis tick interval.
2. **Generate Schedule:** Upon clicking “Generate Schedule,” StaggR calculates the start time for every operation of every sample. In the optimization mode, it iteratively tests increasing staggering intervals until it finds the shortest interval that results in no temporal overlap of manual tasks.
3. **Visualize and Export:** The results are presented in three tabs:
  • **Gantt Chart:** A comprehensive visualization of the entire experimental timeline showing the hands-on time for each sample and operation. It also includes a “Hands-On Time” track that aggregates all manual tasks, clearly indicating when the researcher is busy, free, or has scheduling conflicts (in manual mode).
  • **Schedule by Time:** A chronological, time-stamped list of every action to be performed.
  • **Schedule by Sample:** A table detailing the start time of each operation for each sample.
4. **Execute:** StaggR provides multiple views that can assist in the execution of charted workflows. The “Live Time Course” tab helps you execute the plan, providing live updates as operations become due. Under the “Schedule by Time” tab, the chronological table provides live updates for constant clarity in the execution of the past, current, and forthcoming operations. The “Schedule by Sample” tab shows the timing for processing grouped by each sample. All outputs, including the plot (PDF, PNG, etc.), parameter set, and data tables can be downloaded individually as a zip report for record-keeping and sharing workflows.

## Use cases

To demonstrate the utility of StaggR, I provide three pre-loaded protocols that exemplify setups common in laboratory work. These use cases can be directly loaded from the “About” tab and act as templates for workflow design with minimal modification.

### Case #1 - Formaldehyde fixation (Highly parallel short incubations)

Fixatives such as formaldehyde crosslink proteins and nucleic acids to preserve their cellular state for applications such as imaging, immunoprecipitation, and mass spectrometry. However, over- and underfixation can easily introduce artifacts that affect reproducibility ( Baranello et al., 2016; Schnell et al., 2012). In this use case, the user has a 12-well dish of coverslips bearing cells of different genetic backgrounds. Each coverslip will receive a 60-minute drug or mock (vehicle) treatment after which the cells will be prepared for confocal imaging targeting a protein of interest. They plan to fix each coverslip for 15 min. The user estimates that it will take 0.5 minutes to add the drug, but each sample will require ~5 min to wash and start the fixation, and another 5 min to wash and halt the fixation.

**Figure 2.**
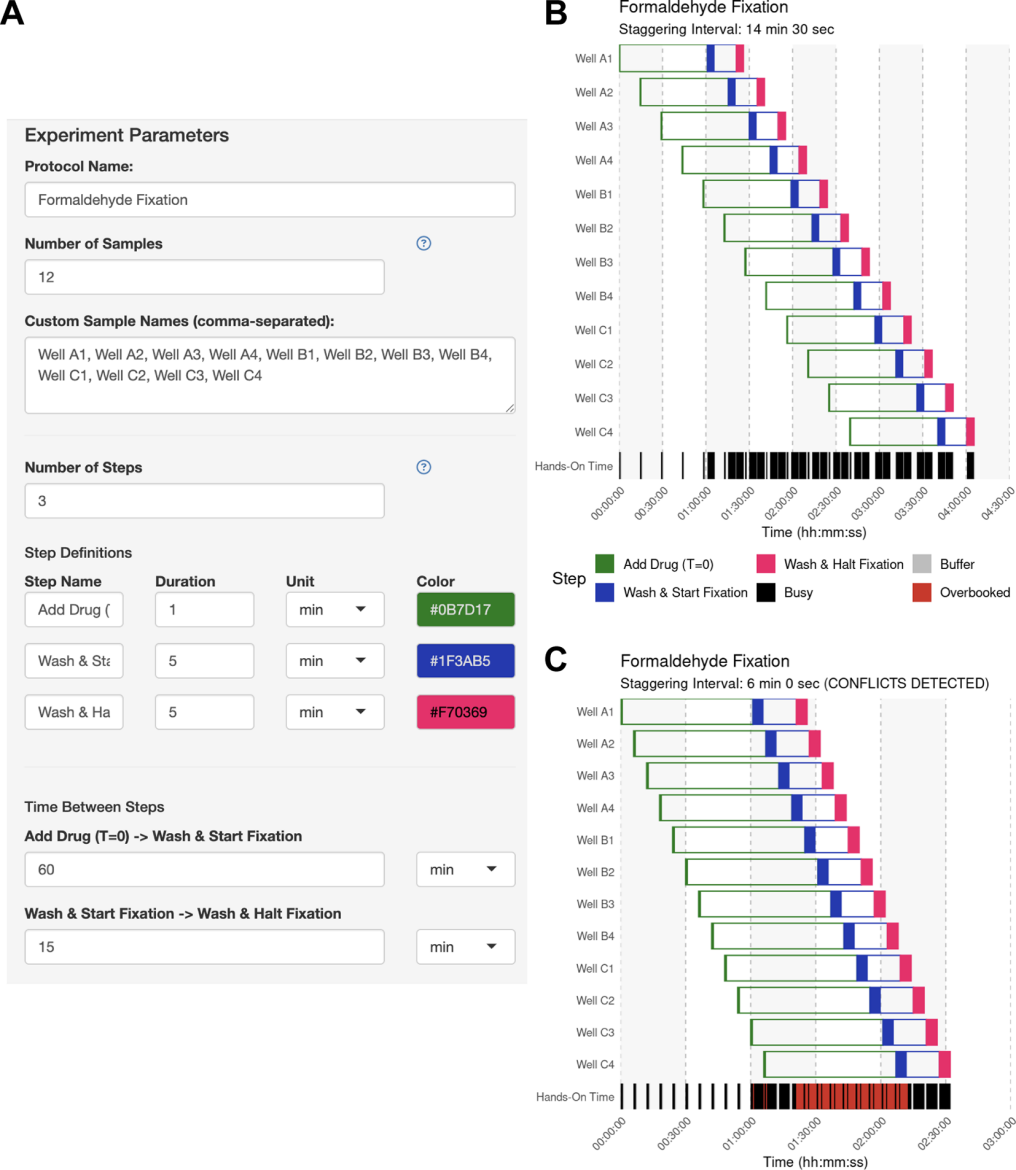
Use Case #1 – Experiment Parameter Input. (A) The experiment parameter side panel is used to define the protocol. Users input sample descriptions and define each protocol step, including its name, color, hands-on duration, and the subsequent inter-step interval (wait time). In this example, each sample receives a 60-minute drug treatment followed by a 15-minute fixation. (B) Automatically identified interval for Use Case #1. Upon generating the schedule, the main panel of StaggR updates with a Gantt chart displaying a viable workflow, minimizing the staggering interval with the given constraints. The “Hands-On Time” row indicates periods that will require the user’s attention and periods where the user is expected to be unoccupied. (C) Manually defined interval for Use Case #1. Users may also provide a pre-defined staggering interval that may or may not produce a viable workflow. Conflicts between steps are immediately apparent and visualized in the “Hands-on Time” row.

The user opens the app and defines the samples and operations used to process each sample, as well as the hands-on duration to execute each operation using the side bar ( Figure 2A). The user then clicks “Generate Schedule” and discovers that a 14-minute and 30-second staggering would be the best solution if they desired to handle each sample individually ( Figure 2B). By enabling the manual mode and inputting a 6-minute interval, the user can immediately visualize the resulting scheduling conflicts ( Figure 2C). This application of StaggR highlights its utility in allowing the exploration of viable and inviable workflows.

### Case #2 – RNA metabolic labeling (Simple time courses executed in parallel)

In this use case, the user wishes to track genome-wide RNA synthesis and decay kinetics in yeast cells after depleting a transcription factor. To do this, they will add 4-thiouracil (4tU), which metabolically labels newly synthesized transcripts, allowing for the measurement of RNA synthesis and decay kinetics. The collection of RNA for 4tU-sequencing experiments ( Barrass et al., 2015), provides genome-wide measurements of RNA production and degradation for every gene. However, this type of experiment is critically sensitive to the labeling time. For their experiment, the user will harvest cell pellets after 5, 15, 30, and 60 min of RNA labeling. They wish to perform these experiments in biological triplicate across four cell lines.

**Figure 3.**
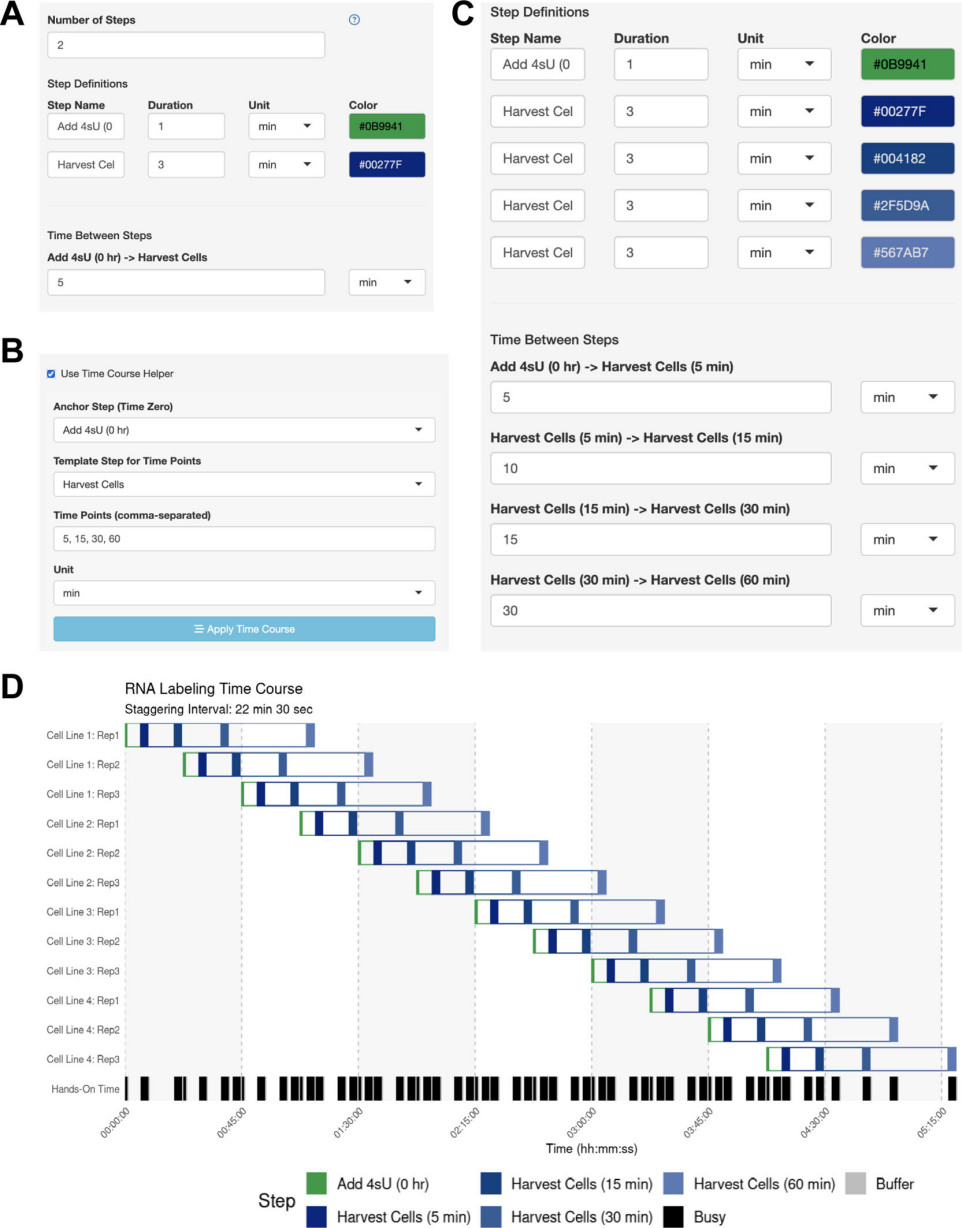
Use Case #2 – Time Course Helper Function. (A-C) Using the Time Course Helper. To use the Time Course Helper, the user will (A) define the “Anchoring” step for initiating the time course and the “Template” step to be repeated throughout (e.g., quench a reaction or harvest a sample), (B) activate the time course helper panel to select the appropriate steps and input the time points of the time course, and (C) apply the time course to automatically insert the time-stamped template steps with the appropriate inter-step interval. (D) Automatically identified interval for Use Case #2. Gantt chart illustrating the automatically-generated interval for Use Case #2.

The user wants to know if they should schedule their experiments across multiple days, or if they can perform 12 independent time courses in parallel. The user performs a dry run of the experiment and thinks that they can initiate the 4tU treatment within 1 min and harvest the cells within 3 min. The user simply defines the anchor step (adding 4tU) and harvest step in the side bar ( Figure 3A) and enables the Time Course Helper, in which they specify the harvesting time points of 5, 15, 30, and 60 minutes after 4tU addition ( Figure 3B). After applying the time course, the step parameters were automatically updated ( Figure 3C), and the user immediately generates their optimal schedule with a 22-minute 30-second staggering period ( Figure 3D). Here, the user leveraged StaggR to quickly and easily produce a viable time course and execute a procedure in one day, which might otherwise have taken several days.

### Case #3 – Sequential drug treatment followed by time course harvest (Complex, multi-sample workflow)

This example outlines a complex workflow to process cultures of human K562 chronic myeloid leukemia cells, in which a protein of interest has been engineered to contain an auxin-inducible degron tag ( Nishimura et al., 2009). The user wishes to examine how cells respond to DNA damage when the protein of interest has been depleted. The objective is to deplete the protein for 4 h with the drug auxin, and then challenge the cells with hydroxyurea (HU), an agent that promotes the accumulation of DNA damage ( Yarbro, 1992). Over 4 h, the user plans to collect protein extracts to probe for levels of γH2AX, a marker of the DNA damage response ( Mah et al., 2010) at time points of 0.5, 1, 2, and 4 h. The user plans to perform this experiment on two cell lines, but each cell line requires four conditions (+auxin/+HU, -auxin/+HU, +auxin/-HU, and-auxin/-HU) for a total of eight independent time courses. They estimated 30 s to add each drug, and 6 min to harvest at each time point.

**Figure 4.**
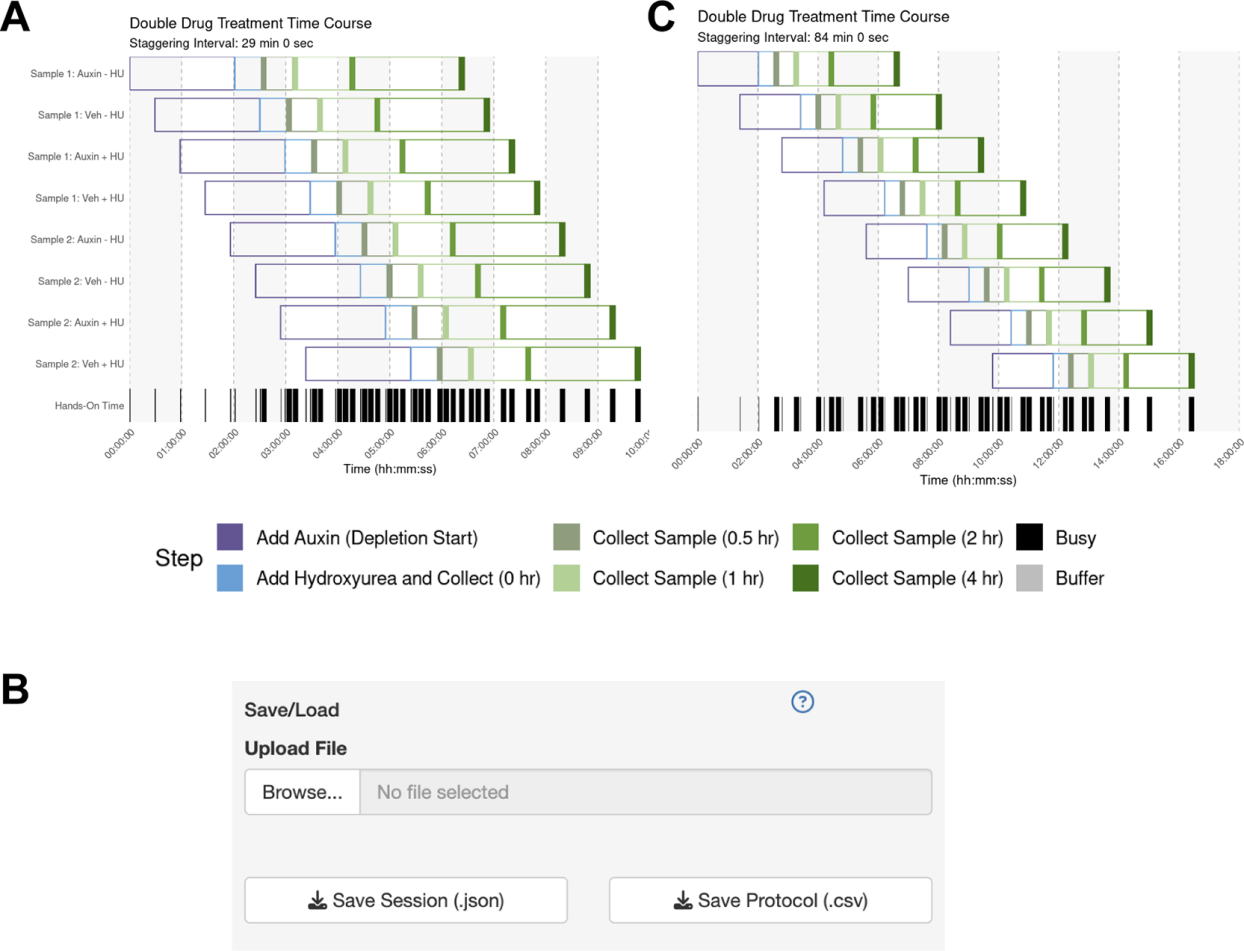
Use Case #3 – Adapting a Complex Workflow for Different Users. (A) Gantt chart showing the optimized schedule for a complex workflow assuming a 6-minute harvest time, resulting in a 28-minute staggering interval. (B) The UI allows the protocol to be saved and shared with a trainee. (C) When the trainee adjusts the harvest duration to their estimated 10 minutes, the regenerated schedule shows that a conflict-free workflow now requires a much longer 84-minute staggering interval, highlighting a potential bottleneck.

**Figure 5.**
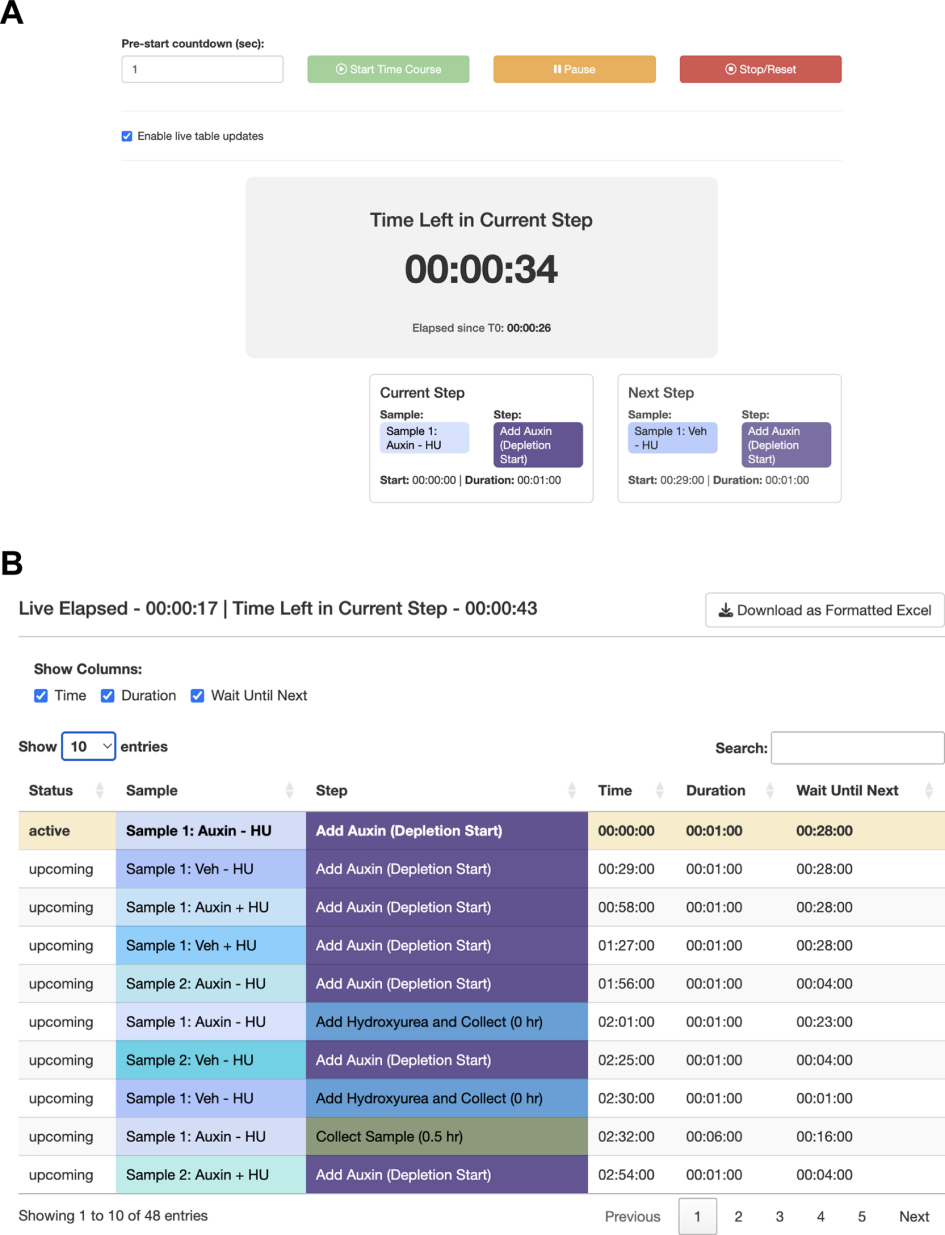
Use Case #3 – Executing the planned workflow with live guidance. StaggR provides live guidance for executing a planned workflow. (A) The timer counts down to the start of the next required hands-on action for any sample. (B) The “Schedule by Time” table provides a chronological list of all steps, with live highlighting to track progression through the protocol.

The user sets up their sample parameters using the Time Course Helper, as in Case #2. After generating the schedule, they find that a 28-minute staggering would work ( Figure 4A). Their trainee wishes to perform a similar protocol, so the mentor downloads the session to share as a.json file ( Figure 4B) which their trainee uploads into their own StaggR instance. However, after a dry run, the trainee estimates that it would take approximately 10 minutes to run the harvest themselves. Inputting 10 min for each harvest and re-generating the schedule shows that in this case, the workflow would require an 84-minute stagger between each sample to execute ( Figure 4C). The trainee decides that they will need to either perform a faster harvesting step or split the experiment over multiple days. The trainee practices the harvest procedure to reduce the time to 6 min per step, loads the original workflow, and navigates to the Live Time Course tab. Here, they initiate the timer to show well-telegraphed step-by-step instructions for sample processing ( Figure 5). Using StaggR, the trainee avoids what would have been an inevitable and critical error in executing this complex workflow.

## Conclusion

StaggR reduces the cognitive load when managing multiple samples with identical treatment schedules by compressing the planning process. This enables the rapid exploration of alternative workflows so that users can quickly assess what is feasible, where conflicts might arise, and where natural breaks occur. This is particularly helpful when protocols are transferred between labs or users, as new execution-time constraints can introduce scheduling conflicts. Sharing protocols and sessions with StaggR makes such conflicts readily apparent and easily preventable. StaggR also boosts the laboratory throughput by minimizing the time required to handle batches of samples.

Many strategies exist to address the FSSP, such as Genetic Algorithms, Simulated Annealing, and Tabu Search ( Komaki et al., 2018). StaggR is not designed to present a novel solution to the FSSP but to provide a convenient platform for experimental design and execution. Future versions of StaggR may implement more nuanced FSSP strategies that make fewer assumptions regarding the homogeneity of each treatment schedule and more efficiently search the space of potential treatment regimes. Nonetheless, as a standalone planning and execution tool, StaggR reduces cognitive barriers to provide immediate value for any lab that aims to perform time-sensitive assays in parallel.

By removing the guesswork and potential error in scheduling identical procedures with asynchronous start times, StaggR can empower researchers from diverse fields to perform high-throughput experiments with greater confidence, efficiency, and reproducibility. Its open source and interactive nature makes it an ideal and broadly applicable tool for planning novel experiments and training personnel on established laboratory workflows.

### Software availability

• **Web Application:** StaggR is available for immediate use at https://alexfrancette.shinyapps.io/staggr/
• **Source code available from:** The complete R source code is available on GitHub: https://github.com/amfrancette/StaggR
• **Archived source code at time of publication**: https://doi.org/10.5281/zenodo.16843661
• **License:** StaggR is distributed under the MIT License.

## Data Availability

• All data underlying the results are available as part of the article and no additional source data are required.
