# Supplementary material for: StaggR: an interactive R/Shiny application for planning and visualizing staggered experimental protocols
Source: F1000Res. 2026 Jan 13;14:927. Originally published 2025 Sep 15. [Version 3] doi: 10.12688/f1000research.168987.3 (PMC12976608; doi:10.12688/f1000research.168987.3)
Supplement: Peer review history

# Reviewer report: Reviewer response for version 3

**DOI:** 10.5256/f1000research.194752.r460176

**Published:** 2026-03-10

**Authors:** Eudald Correig-Fraga

**Affiliations:** 1 Universitat Rovira i Virgili, Tarragona, Spain; 2 Reserach Department, Innovamat Education, Sant Cugat del Vallès, Catalonia, Spain

This paper presents a software tool to organize staggered experiments in a simple yet useful way. The motivation is well explained, and the methods are clear. The source code is provided, together with a link to an instance of the program hosted by the author himself. The case examples already loaded on the app are a very nice addition by the author to understand how to start with the tool.

 A few comments:

 Manuscript:

 - I feel like the first and second paragraph were unadvertedly separated? 

 - It's not completely clear to me what's the state of the art to solve this issue. The author says "quickly become intractable to plan manually", and then present solutions for machining operations, but people are planning now; are they using commercial software (if so, which one), or very complex and unmaintainable spreadsheets? this comparison again should be raised in the conclusion

 - the citations are a bit haphazard: why is dplyr or ggplot cited, but shiny is not? Or Tabu Search, but not Simulated Annealing. 

 - There is a "Core logic" title that I don't know what refers to.

 - I don't think the performance section is relevant for such a simple tool. In any case, if it's added, I'm more interested in how long does it take for the tool to give me a solution, than the amount of RAM it uses.

 Code:

 - Since the author started using tidyverse (dplyr), I would recommend go all the way, and do all the manipulations in tidyverse.

 - There are benchmark tests, but not logic tests; it would be nice to add them.

 - The code should be formatted consistently (for example, in RStudio, doing Ctrl+A, then Ctrl + Shift + A).

Are the conclusions about the tool and its performance adequately supported by the findings presented in the article?

Yes

Is the rationale for developing the new software tool clearly explained?

Yes

Is the description of the software tool technically sound?

Yes

Are sufficient details of the code, methods and analysis (if applicable) provided to allow replication of the software development and its use by others?

Yes

Is sufficient information provided to allow interpretation of the expected output datasets and any results generated using the tool?

Yes

Reviewer Expertise:

Statistics, Programming, Computational neuroscience.

I confirm that I have read this submission and believe that I have an appropriate level of expertise to confirm that it is of an acceptable scientific standard.

# Reviewer report: Reviewer response for version 3

**DOI:** 10.5256/f1000research.194752.r449912

**Published:** 2026-01-15

**Authors:** Charuka D Wickramasinghe

**Affiliations:** 1 Oncology, Wayne State University School of Medicine (Ringgold ID: 12267), Detroit, Michigan, USA; 2 Wayne State University, Detroit, Michigan, USA

No further comments to make. I Approved this.

Are the conclusions about the tool and its performance adequately supported by the findings presented in the article?

Yes

Is the rationale for developing the new software tool clearly explained?

Yes

Is the description of the software tool technically sound?

Yes

Are sufficient details of the code, methods and analysis (if applicable) provided to allow replication of the software development and its use by others?

Yes

Is sufficient information provided to allow interpretation of the expected output datasets and any results generated using the tool?

Yes

Reviewer Expertise:

Bioinformatics; Pharmacology; Applied Mathematics; Programming; Machine Learning

I confirm that I have read this submission and believe that I have an appropriate level of expertise to confirm that it is of an acceptable scientific standard.

# Reviewer report: Reviewer response for version 2

**DOI:** 10.5256/f1000research.193274.r442762

**Published:** 2026-01-02

**Authors:** Charuka D Wickramasinghe

**Affiliations:** 1 Oncology, Wayne State University School of Medicine (Ringgold ID: 12267), Detroit, Michigan, USA; 2 Wayne State University, Detroit, Michigan, USA

This article presents **StaggR**, an interactive web application that calculates and visualizes compatible staggering intervals for parallelized execution of identical processing workflows. StaggR provides a user-friendly interface for defining protocol operations, durations, and wait times. Overall, the article has a strong potential for indexing after the following corrections. A few bugs were identified in the R Shiny code.

1. **(Major)** The R Shiny application is disconnected from the server when the number of samples or sample names is changed.
2. **(Major)** Under the *“About”* tab, the R Shiny application is disconnected from the server when the example protocols are clicked.
3. **(Minor)** The first three question mark (“?”) icons in the left panel of the user interface do not display any information. Adding appropriate descriptions for these three icons is recommended.
4. **(Minor – optional)** Adding a downloadable sample input file next to the file upload tab is recommended to help users better understand how the application works.
5. **(Minor – optional)** Adding a short video demonstration illustrating the functionality of the application is recommended.

Are the conclusions about the tool and its performance adequately supported by the findings presented in the article?

Yes

Is the rationale for developing the new software tool clearly explained?

Yes

Is the description of the software tool technically sound?

Yes

Are sufficient details of the code, methods and analysis (if applicable) provided to allow replication of the software development and its use by others?

Yes

Is sufficient information provided to allow interpretation of the expected output datasets and any results generated using the tool?

Yes

Reviewer Expertise:

Bioinformatics; Pharmacology; Applied Mathematics; Programming; Machine Learning

I confirm that I have read this submission and believe that I have an appropriate level of expertise to confirm that it is of an acceptable scientific standard, however I have significant reservations, as outlined above.

## References

[1] : SpatialCNS ‐PBPK : An R/Shiny Web‐Based Application for Physiologically Based Pharmacokinetic Modeling of Spatial Pharmacokinetics in the Human Central Nervous System and Brain Tumors. *CPT: Pharmacometrics & Systems Pharmacology* .2025;14(5) : 10.1002/psp4.70026 864-880 10.1002/psp4.70026 PMC1207222840183989

# Response

**Published:** 2026-01-06

**Authors:** Alex Francette

**Affiliations:** Cell Biology and Physiology, Washington University in St Louis Department of Cell Biology and Physiology, St. Louis, Missouri, USA

I thank you for identifying several areas for improvement for the app. I have addressed your comments as follows:

 Reviewer 2, Comments 1,2,3: Fixed bugs introduced by transferring core scheduling logic outside of the Shiny server function. The app no longer disconnects when changing sample names/count or clicking example protocols, and the first three "?" icons now display appropriate information.

 Reviewer 2, Comment 4: Added a "Download Sample Session (.json)" button next to the file upload tab to make the app more user-friendly.

 Reviewer 2, Comment 5: I believe a video demonstration is a good idea, and I will strongly consider creating one to include in future updates of the app.

# Reviewer report: Reviewer response for version 1

**DOI:** 10.5256/f1000research.186237.r428843

**Published:** 2025-11-25

**Authors:** Thomas Darde

**Affiliations:** 1 SciLicium, 10 Rue de La Sauvaie, Rennes, France

This manuscript presents StaggR, an interactive and open-source R/Shiny application designed to assist researchers in planning, visualizing, and executing staggered experimental workflows, particularly for time-sensitive molecular biology assays. The complexity of coordinating multiple samples with identical but offset treatment sequences is a well-known bottleneck in wet-lab research. StaggR provides a practical solution that integrates workflow definition, conflict detection, Gantt chart visualization, and a live execution timer. The tool is accessible, fully open-source, and addresses a reproducibility challenge often overlooked in workflow design. The manuscript is clearly written and the tool appears directly usable. Overall, the article has strong potential for indexed once several key revisions are addressed.

 1. (Major) Lack of performance and scalability evaluation

 The scheduling algorithm is described as a simple heuristic. However, the manuscript provides no benchmarks evaluating:

 - runtime as sample number increases,

 - scalability to large workflows,

 - memory/time constraints during optimization.

 A small benchmarking section or supplementary figure would greatly strengthen the work.

 2. (Minor)Insufficient description of failure modes

 The manuscript does not clearly explain:

 - how StaggR behaves when no conflict-free solution exists,

 - how users are alerted to impossibility,

 - whether alternative suggestions are offered.

 3.(Minor) The scheduling algorithm needs more technical depth

 The manuscript briefly references the Flow Shop Scheduling Problem (FSSP), but does not justify the heuristic relative to classical approaches. A clearer section explaining constraints, assumptions, and limitations is required.

 4. (Major) Add a conceptual software architecture diagram

 The paper includes only dashboard screenshots. A diagram of the internal logic and components would enhance clarity.

 5. (Minor) Clarify ambiguous parameters

 Explain granularity, buffer time, and unit consistency with examples.

 6.(Minor) Improve consistency in terminology

 Standardize hands-on, wait time, buffer, etc.

 7.(Minor) Discuss limits of heterogeneous protocols

 The tool assumes identical steps across samples; this should be clearer.

Are the conclusions about the tool and its performance adequately supported by the findings presented in the article?

Yes

Is the rationale for developing the new software tool clearly explained?

Yes

Is the description of the software tool technically sound?

Yes

Are sufficient details of the code, methods and analysis (if applicable) provided to allow replication of the software development and its use by others?

Yes

Is sufficient information provided to allow interpretation of the expected output datasets and any results generated using the tool?

Partly

Reviewer Expertise:

Bioinformatics; Toxicology; Toxicogenomics, Bulk RNA-seq

I confirm that I have read this submission and believe that I have an appropriate level of expertise to confirm that it is of an acceptable scientific standard, however I have significant reservations, as outlined above.

# Response

**Published:** 2025-12-08

**Authors:** Alex Francette

**Affiliations:** Cell Biology and Physiology, Washington University in St Louis Department of Cell Biology and Physiology, St. Louis, Missouri, USA

I thank you, reviewer, for your positive comments and insightful suggestions. I have made several changes to the main manuscript to incorporate your feedback. I will address your comments point by point.

**Reviewer 1, Comment 1: (Major) Lack of performance and scalability evaluation**

**Response: **I have added a benchmarking section to the manuscript to evaluate the performance and scalability of the scheduling algorithm used in StaggR. This section includes analyses of runtime as the number of samples increases, scalability to larger workflows, and memory/time constraints during optimization. The results are presented in a new figure (Figure 7) and discussed in the main text.

**Reviewer 1, Comment 2: (Minor) Insufficient description of failure modes**

**Response: **I have expanded the manuscript to include more detailed descriptions of how StaggR handles situations where no conflict-free solution exists. The manuscript now explains how users are alerted to such impossibilities through error messages in the application interface. Additionally, I have clarified that while StaggR offers general suggestions for adjusting protocol parameters, it does not currently provide alternative scheduling solutions when a conflict-free schedule cannot be generated.

**Reviewer 1, Comment 3: (Minor) The scheduling algorithm needs more technical depth**

**Response: **I have revised the manuscript to provide a more in-depth explanation of the scheduling algorithm used in StaggR. This includes a discussion of the constraints, assumptions, and limitations of the heuristic approach relative to classical scheduling methods emphasizing the prioritization of ease of use and flexibility for researchers without computational training.

**Reviewer 1, Comment 4: (Major) Add a conceptual software architecture diagram**

**Response: **I have added a new figure (Figure 2) that provides a conceptual software architecture diagram of StaggR. This diagram illustrates the internal logic and components of the application, enhancing clarity for readers.

**Reviewer 1, Comment 5-6: (Minor) Clarify ambiguous parameters and improve consistency in terminology**

**Response: **I have expanded descriptions of "granularity," "buffer time," "hands-on time," and "wait time." Each term is explained with examples to ensure clarity. Units of time have been defaulted to minutes for consistency. Additionally, I have reviewed the manuscript to ensure consistent usage of these terms.

**Reviewer 1, Comment 7: (Minor) Discuss limits of heterogeneous protocols**

**Response: **I have clarified in the manuscript that StaggR currently assumes identical steps across all samples in a protocol. I have discussed the implications of this assumption and noted that future versions of the tool may explore support for heterogeneous protocols.

 I believe these modifications have greatly strengthened the messaging, clarity, and rigor of this work.
